# Influence of the ferric uptake regulator (Fur) protein on pathogenicity in *Pectobacterium carotovorum* subsp. *brasiliense*

**DOI:** 10.1371/journal.pone.0177647

**Published:** 2017-05-17

**Authors:** Collins Kipngetich Tanui, Divine Yutefar Shyntum, Stefan Louis Priem, Jacques Theron, Lucy Novungayo Moleleki

**Affiliations:** 1 Forestry, Agriculture and Biotechnology Institute, University of Pretoria, Pretoria, South Africa; 2 Department of Microbiology and Plant Pathology, University of Pretoria, Pretoria, South Africa; Niels Bohr Institute, DENMARK

## Abstract

Iron is an important nutrient for the survival and growth of many organisms. In order to survive, iron uptake from the environment must be strictly regulated and maintained to avoid iron toxicity. The ferric uptake regulator protein (Fur) regulates genes involved in iron homeostasis in many bacteria, including phytopathogens. However, to date, the role played by Fur in the biology of *Pectobacterium carotovorum* subsp. *brasiliense* (*Pcb1692*), an important pathogen of potatoes, has not yet been studied. To this end, we used the lambda recombineering method to generate a *fur* mutant strain of *Pcb1692* and assessed the virulence and fitness of the mutant strain. The results showed that production of siderophores in *Pcb*1692Δ*fur* increased compared to the *Pcb1692* wild-type and the complemented strain *Pcb*1692Δ*fur*-*pfur*. However, production of N-acyl homoserine lactone (AHLs), biofilm formation, exopolysaccharide (EPS) production, virulence on potato tubers and swimming motility, were all significantly decreased in *Pcb*1692Δ*fur* compared to the wild-type and complemented *Pcb*1692Δ*fur*-p*fur* strains. The *Pcb*1692Δ*fur* mutant also demonstrated significant sensitivity to oxidative stress when exposed to H_2_O_2_. Consistent with phenotypic results, qRT-PCR results demonstrated that Fur down-regulates genes which encode proteins associated with: iron uptake (HasA-extracellular heme-binding protein and Ferrodoxin-AED-0004132), stress response (SodC-superoxide dismutase), plant cell wall degrading enzymes (PrtA and CelV) and motility (FlhC and MotA). We conclude that the ferric uptake regulator protein (Fur) of *Pcb1692* regulates traits that are important to host-pathogens interactions.

## Introduction

*Pectobacterium carotovorum* subsp. *brasiliense* (*Pcb*) is a member of the soft rot Enterobacteriaceae (SRE), which consist of members of the *Pectobacterium* and *Dickeya* genera [1]. This pathogen is the major causal agent of blackleg and soft rot disease in potato stems and tubers, respectively, in the field and during post-harvest [2]. *Pcb* strains have been reported in many different countries where they have been shown to cause significant losses to the potato industry and a wide variety of crops in many countries, including Brazil, Kenya, New Zealand and South Africa [2–5]. This pathogen has been identified to be more virulent compared to other *Pectobacterium* species [2]. Like other SREs, *Pectobacterium* species use a variety of virulence determinants to adapt, colonize and cause disease in plants. Some of these virulence factors include, amongst other, density-dependent cell-cell communication mediated by acyl homoserine lactones (AHLs), secretion systems, plant cell wall-degrading enzymes (PCWDEs), adhesion, biofilm formation, motility, siderophores and chemotaxis [6–12].

During the host invasion process, pathogenic bacteria encounter different environmental conditions, of which iron limitation and reactive oxygen species produced by the host plant are major factors limiting the ability of the bacteria to spread and colonize the host [13, 14]). Therefore, bacteria must tightly regulate iron uptake and deal with changes occurring during redox conditions [14]. In effect, the host withholds iron, depriving the pathogen of iron and thus limiting its ability to colonize the host plant. However, bacterial pathogens have developed means of acquiring scarce iron through siderophore production [14, 15]. For example, the roles of high-affinity iron siderophores such as chrysobactin and achromobactin in iron uptake during *Dickeya dandantii* (formerly *Erwinia chrysanthemi*) colonization of potato tubers, has been demonstrated [16]. In this respect, studies by Franza and colleagues demonstrated that mutagenesis of genes encoding chrysobactin and achromobactin resulted in impaired symptom initiation, suggesting an inability of the pathogen to survive within host intracellular spaces [15, 16].

In addition to the above, SREs can use their PCWDEs to rupture plant cells, thus making nutrients more accessible to the bacteria [17]. In fact, regulation of PCDWEs is often coupled with iron acquisition in *Dickeya dandantii* [15]. Once nutrients are released, this presents a free-for-all ‘microbes’ situation. Hence, the fittest bacteria will try to assimilate all available iron to themselves. In this respect, *Pcb* has been shown to use a vast number of ‘antibacterial’ strategies to inhibit growth of some members of the SRE and other bacteria, both *in vitro* as well as in potato tubers [18]. It can thus be hypothesized that these arsenals of antibacterial factors are necessary for nutrient and/or iron acquisition. In many bacteria, iron homeostasis is regulated by the ferric uptake regulator protein encoded by the *fur* gene. Notably, mutation of the *fur* gene in *Pseudomonas aeruginosa* resulted in defects in iron uptake and reduced virulence [19].

The Fur protein is a transcriptional repressor with the ferrous ion (Fe(II)) as a co-repressor [20, 21]. When levels of iron exceed those required for cellular functions, Fur represses further iron uptake in order to prevent iron overload and toxicity. In many pathogenic bacteria, Fur protein is also implicated in the regulations of virulence determinants unrelated to iron transport such as PCWDEs [16]. In the presence of ferrous ion, Fur binds to the ‘fur box’, a conserved DNA sequence located within the promoter regions of iron-regulated genes [22–25]. The “fur box” in *Escherichia coli* is predicted to have a consensus sequence of GAT AAT GAT AAT CAT TAT C. However, this exact sequence is rarely found in other prokaryotes. In fact, it has been suggested that the minimum recognition for iron binding is GAT AAT and in prokaryotes the exact mode of recognition is still relatively unknown [26]. Furthermore, the ‘fur box’ can be highly variable even in genes within the fur regulon of a given bacterium. For example, the predicted fur boxes of the *pelD* and *pelE* genes in *Erwinia chrysanthemi* 3937 are GAT AAA ATT AAT CAG CCT C and ATT AAT AAA AAC CAT TGT C, respectively [16, 27].

In this study, the *Pectobacterium carotovorum* subsp. *brasiliense fur* gene homolog was identified, a *fur* mutant strain generated using methods previously described by Datsenko and Wanner [28] and functionally characterized. Its role in virulence and its effect on *Pcb1692* virulence factors, including biofilm formation, production of acyl homoserine lactone, swimming motility, extracellular polysaccharide and extracellular enzymes production, was also investigated.

## Materials and methods

### Strains and growth conditions

All bacterial strains and plasmids used in this study are listed in Table 1. Bacterial strains were grown on nutrient agar or in liquid Luria-Bertani (LB) broth and in M9 minimum medium at 37^°^C [29]. Where necessary, growth media were supplemented with either100 μg/ml Ampicillin (Sigma-Aldrich), 50 μg/ml Kanamycin (Sigma-Aldrich) or 10 mM MnSO_4_(Sigma-Aldrich).

**Table 1:** Bacterial strains and plasmids used in this study.

Bacterial strains	Description	Sources
*Pectobacterium carotovorum* subsp. *brasiliense* 1692 *(Pcb1692)*	Initially isolated from potato in Brazil, sequenced strain	[2]
*Pcb*1692Δ*fur*	*Pcb*1692Δ*fur*, Kan^r^	This study
*Pcb*1692Δ*fur-pfur*	*Pcb*1692Δ*fur* expressing the *fur* gene from the Trc99A plasmid; Kan^r^, Amp^r^	This study
*Pcb*1692Δ*expI*	*Pcb*1692Δ*expI*, Kan^r^	[7]
*Chromobacterium violaceum CV026*	AHL reporter strain	[25]
**Plasmids**		
pKD4	Plasmid containing a Kan^r^ cassette	[28]
pKD46	Plasmid expressing the lambda red genes	[28]
pTrc99A	Bacterial expression vector	[30]
pTrc99A-*fur*	Bacterial expression vector containing the *fur* gene insert, Amp^r^	This Study

### *Generation of a Pectobacterium carotovorum* subsp. *brasiliense fur* mutant strain

The *fur* gene of *Pectobacterium atrosepticum* (*ECA1329*) was used as a query to identify the *fur* homolog in *Pcb1692*. Using the BLASTN alignment tool available on the ASAP database, we identified the *fur* homolog in *Pcb1692* (PcarbP_010200018626) on contig 00060 (coordinates 25375–25824) with 100% identical to *ECA1329*. The *Pcb*1692Δ*fur* mutant strain was generated using the strategy described previously by Datsenko and Wanner [28] and is indicated in S1 Fig. In brief, the upstream and downstream regions flanking the *Pcb1692 fur* gene (approx. 1200bp) were amplified by polymerase chain reaction (PCR). The kanamycin resistance gene was PCR amplified from plasmid pKD4. The three amplicons were fused by overlap extension PCR to produce a gene disruption cassette, as described previously [31]. The fused PCR product was then electroporated into *Pcb1692* harboring pKD46 and transformants were selected on nutrient agar supplemented with 50 μg/ml kanamycin. The list of primers used in this study is provided in Table 2.The HiFi HotStart PCR Kit (KAPA Biosystems) was used in all PCR reactions.The PCR thermal cycling conditions were set as follow: initial denaturation at 95^°^C for 3 min, followed by 30 cycles of denaturation at 98^°^C for 30 s, annealing at 60–64^°^C for 15 s (depending on the primer set), extension at 72^°^C for 3 min and a final extension at 72^°^C for 5 min. The integrity of the *Pcb*1692Δ*fur* mutant strain was confirmed by PCR analyses, nucleotide sequencing and Southern blot analysis (results not shown).

**Table 2:** Primers used in this study.

Primer name	Sequence (5’-3’)	Length (bp)
	Mutagenesis primers	
Fur–F	CGATCAACTGCACGCTTATGC	21
Fur-R	GAATAGTAATGAGCCATTACGC	22
Test–F	AAGATCTGGCGTCCGGTAAGC	21
Test -R	TCATTCTGAGACTAAACGCACC	22
Furkan -R	CGAAGCAGCTCCAGCCTACACATCAACACGATAAATCGACCGC	43
Furkan -F	CTAAGGAGGATATTCATATGTTAATCCTGTTGCTTACTTATC	42
Kan–F	GCGGTCGATTTATCGTGTTGATGTGTAGGCTGGAGCTGCTTCG	43
Kan -R	GATAAGTAAGCAACAGGATTAACATATGAATATCCTCCTTAG	42
Comp -F	GATTTATCGTGTTGAATCGTC	21
Comp -R	GCCATTTCGGCTCTGATAATC	21
Ffh–F	TGGCAAGCCAATTAAATTCC	20
Ffh–R	TCCAGGAAGTCGGTCAAATC	20
Fer-F	AGACCCATCATCGGTAGCAC	20
Fer -R	CTCATCTTCCGCAAAGAAGC	20
HasA–F	ATTTACGGCCTGATGAGTGG	20
HasA–R	AACGACGTCAACCACGGTAT	20
CelV–F	CGTTAAACCGGAACCAACTG	20
CelV -R	AACCACCGTACTGCCTTTTG	20
Mot A -F	TTGCCTACGGTTTTGTCTCC	20
Mot A -R	ACAGCGTTTTACGACCGAAT	20
SodC- F	TAAATCAGTTCCCGCTCTGG	20
SodC-R	GCCAGAATTGGGTAGGTTGA	20
FlhC -F	ATTGCTGCAAAGGGATGTTC	20
FlhC -R	CCTGTTCATCCAGCAGTTGA	20
PrtA -F	TGACGCGTTGCATTCATTAT	20
PrtA–R	TGCCAAATACATTCGAACCA	20

### Generation of a complemented *Pcb*1692Δ*fur* strain

The *fur* gene and its putative promoter region was PCR amplified from *Pcb1692* using primers Comp-F and Comp-R (Table 2). The amplicon was excised from an agarose gel and purified using the Zymo Clean Gel DNA Recovery Kit (Inqaba Biotec, South Africa) according to the manufacturer’s instructions. The amplicon was cloned into pTrc99A to generate pTrc99A*-fur* (Table 1). The pTrc99A*-fur* plasmid was electroporated into the *Pcb*1692Δ*fur* mutant strain and the transformants (*Pcb*1692Δ*fur-*p*fur*) were selected on agar plates supplemented with 100 μg/ml Ampicillin.

### *In vitro* growth assays

The *in vitro* growth properties of the *Pcb1692*, *Pcb*1692Δ*fur* and *Pcb*1692Δ*fur-*p*fur* strains were assessed by culturing each bacterial strain in M9 minimal medium and liquid LB broth. The cultures were incubated at 37°C for 16 h with agitation at 370 rpm. The optical density at 600 nm (OD_600_) of the overnight cultures was adjusted to an OD_600_ of 0.1, and 1 ml was inoculated into 200 ml of LB broth or M9 minimal medium and grown at 37°C with agitation at 370 rpm. The OD_600_ was recorded every hour for 16h with a Multiskan GO spectrophotometer (Thermo-Scientific). The experiment was performed in triplicates, three independent times.

### Siderophore production

Siderophore production was determined using Chrome Azurol S (CAS) agar plates, as described previously by Louden et al. [32]. Briefly, the *Pcb1692*, *Pcb*1692Δ*fur* and *Pcb*1692Δ*fur-*p*fur* strains were grown in LB broth for 16 h with shaking at 370 rpm. The OD_600_ of the cultures was then adjusted to 1.0,and 50 μl of each strain was spotted onto a CAS agar plate and incubated at 37°C for 48 h. A yellow halo surrounding the inoculation site was taken to indicate production of siderophores. The diameter of each halo was measured in mm. This experiment was performed in triplicates, three independent times.

### Resistance against hydrogen peroxide (H_2_O_2_)

To determine resistance to H_2_O_2_, the *Pcb1692*, *Pcb*1692Δ*fur* and *Pcb*1692Δ*fur-*p*fur* strains were grown in LB broth for 16 h in a shaking incubator at 370 rpm. The OD_600_of each culture was adjusted to 0.4 and 100μl of each bacterial culture was then inoculated into 100 ml of LB broth supplemented with 20 μM H_2_O_2_. The cultures were incubated for at 37^°^Cfor 6h with shaking (370 rpm) and the surviving bacteria were enumerated by serial dilution and plating onto LB agar plates.

### Virulence assays

Surface-sterilized potato tubers (cv. Mondial, a susceptible cultivar) were stabbed to a depth of about 1cm with a sterile pipette tip. A 10-μl aliquot of the *Pcb1692*, *Pcb*1692Δ*fur-*p*fur* and *Pcb*1692Δ*fur* cultures (OD_600_ = 1) was inoculated into the wounded tubers. For the negative controls, sterile 10 mM MgSO_4_ was inoculated into the wounded potato tubers. The inoculated potato tubers were placed in moist plastic bags and incubated at 25^°^C. At 72h post-inoculation, the macerated tissue was scooped and weighed to quantify the extent of tuber maceration by each of the different bacterial strains. This experiment was performed in triplicates, three independent times.

### Detection of N-acyl homoserine lactones (AHLs)

The *Chromobacterium violaceum* (CV026) reporter strain [33] was inoculated into 30 ml of LB broth supplemented with 30 μg/ml kanamycin and grown at 28°C for 16 h with shaking at 270 rpm. A 1-ml aliquot of the overnight CV026 culture was added to 3 ml of filter-sterilized cultures of the *Pcb*1692, *Pcb1692*Δ*expI*, *Pcb*1692Δ*fur* and *Pcb*1692Δ*fur-*p*fur* strains in different Falcons tubes. The *Pcb1692*Δ*expI* strain was used as negative control. Bacterial cultures were then incubated at 28°C for 48 h. A blue colour indicated the production and presence of AHLs. This experiment was performed in triplicates, three independent times.

### Swimming motility assay

Swimming motility assays were performed at 37^°^C on LB agar plates containing 0.3% (w/v) Bacto agar. The *Pcb1692*, *Pcb*1692Δ*fur* and *Pcb*1692Δ*fur-*p*fur* strains were grown overnight in LB broth and the OD_600_ of each culture was then adjusted to 0.5. A sterile toothpick was dipped into each bacterial culture and then spotted in the middle of the LB agar plates. The agar plates used for inoculation of the *Pcb*1692Δ*fur* mutant strain were supplemented with 50 μg/ml kanamycin. The agar plates were incubated at 37°C for 24 h. swimming motility was determined by measuring the halos formed by swimming bacteria after 24 h.

### Biofilm formation assay

Formation of biofilm was analyzed as described previously by Daniel Perez-Mendoza and colleagues with minimal modifications [34]. In brief, the OD_600_ of overnight cultures of *Pcb1692*, *Pcb*1692Δ*fur* and *Pcb*1692Δ*fur-*p*fur* were adjusted to 0.4. Subsequently, 25 μl of each bacterial culture was inoculated into 40 ml of LB broth and incubated at 37°C for 48h with shaking at 130 rpm. The LB broth was aspirated and biofilm that formed on the walls of the 50-ml Erlenmeyer flasks were visualized by washing three times with double distilled water followed by staining with 0.1% (w/v) crystal violet (Sigma-Aldrich) then incubated for 30 min at room temperature. A violet-coloured ring on the inner wall of the flask indicated biofilm formation. Biofilm formation was also quantitatively assayed by measuring the OD_570_ of the stained suspensions in 96-well plates with a spectrophotometer.

### Extracellular enzyme assays

Semi-quantitative analysis of two plant cell wall-degrading enzymes (PCWDEs), cellulase and proteases, were performed as described by Chatterjee and colleagues [35]. Holes were made on assay plates and the bacterial strains were inoculated into these holes. Cellulase assay plates were stained with 0.1% (w/v) Congo red solution (Sigma-Aldrich), incubated for 30 min and then washed several times with 1M NaCl until a clear zone became visible around the holes. After three days of incubation, Protease plates revealed clear zones around the holes without any further treatment. Enzyme activity was semi-quantified based on the diameter of the haloes around the colonies.

### Manganese resistance assay

Resistance to manganese was determined according to the protocol described previously by Hentke [36]. The *Pcb1692*, *Pcb*1692Δ*fur-*p*fur* and *Pcb*1692Δ*fur* strains were grown for 16 h in LB broth and the OD_600_ of each culture was then adjusted to 1.0.The bacterial strains were plated onto LB agar supplemented with 7mM MnSO_4_.H_2_O (Sigma-Aldrich), followed by incubation at 37^°^C for 24 h. Three biological experiments were performed.

### Extracellular polysaccharide (EPS) production determination

EPS production was measured as described previously by Tang *et al*. [37], with minor modifications. Briefly, cultures of *Pcb1692*, *Pcb*1692Δ*fur* and *Pcb*1692Δ*fur-*p*fur* were grown in 100 ml LB broth at 37°C for 72 h to an OD_600_ of 2.5. The cultures were then transferred into 50-ml Falcon tubes and centrifuged at 10000 rpm for 10 min. EPS was collected from the supernatant by precipitation with 96% ethanol, dried at 37°C for 3 h and weighed. The experiment was performed in triplicates.

### qRT-PCR assays

Bacterial cultures were grown overnight in LB broth and the cells harvested by centrifugation at 14000 rpm for 1 min. The supernatant was carefully discarded and the bacterial cells were suspended in RNA stabilization buffer (Qiagen, Hilden, Germany). Total RNA was extracted with the RNeasy mini kit (Qiagen, Hilden, Germany) following the manufacturer’s instructions. DNaseI (Qiagen, Hilden, Germany) was used to remove contaminating genomic DNA from the total RNA samples. The concentration of the total RNA was determined using a NanoDrop1000 spectrophotometer (NanoDrop® Technologies, Wilmington, DE). First-strand cDNA was synthesized from 1 μg of the total RNA samples using the Superscript VI First-Strand Synthesis system kit (Invitrogen).The list of primers used for qRT-PCR is provided in Table 2. The genes targeted for qRT-PCR included: *prtA* (protease), *celV* (cellulose),*AED-0004132* (ferredoxin) *hasA* (extracellular heme-binding protein), *motA* (flagellar motor), *flhC* (flagellar transcriptional regulator), *sodC* (copper-zinc superoxide dismutase) and *ffh* (signal recognition particle subunit). The *ffh* gene was used as internal normalization gene (Table 2). The cDNA was used in real-time PCR reactions using a Quantstudio 12 flex thermocycler (Applied Biosystems). Experiments were performed in triplicates and three biological replicates were performed for each gene. Comparative 2^ΔΔct^ method was used to analyze the data.

### Prediction of putative ‘fur boxes’

Genes previously shown to be under Fur regulation in other organism were selected and homologs in *Pcb1692* were identified. Thereafter, using CLC bioinformatics analysis regions upstream of the start codon of each gene sequence were screened for DNA sequences containing the GATAAT signature sequence. These included *pelA*, *expI*, *flhD*, *hasA* and *tonB* among others. The “fur box” consensus was manually aligned with selected *fur* regulated genes to match one of the hexameric repeats. Red boxes were added to indicate all possible alignments within the consensus sequence.

### Statistical analysis

In this study, experiments were performed in triplicate and three independent times. Where applicable, a one-way Analysis of Variance (ANOVA) was performed to determine statistical significance and a *p*-value less than 0.05 (*p*<0.05) was considered to be a statistically significant difference.

## Results

### Construction and characterization of *Pcb*1692Δ*fur* mutant

In order to determine the role of the Fur protein in *Pcb1692*, we generated a *Pcb*1692Δ*fur* mutant using the lambda Recombination technique (S1 and S2 Figs), as described in the Material and Methods section. The integrity of *Pcb*1692Δ*fur* mutant was verified by PCR analyses, nucleotide sequencing and Southern blotting, the results of which confirmed that there was a single insertion of the kanamycin cassette into the genome sequence of *Pcb* resulting in deletion of the *fur* gene. Similarly, the integrity of the complemented *fur* gene (with endogenous promoter) cloned into plasmid Trc99A was also confirmed by nucleotide sequencing and the recombinant plasmid was stably maintained in the *Pcb*1692Δ*fur* mutant strain (results not shown). *In vitro* growth assay demonstrated that deletion of the *Pcb fur* gene does not impair the growth of the mutant strain (results not shown).

### The role of Fur in *Pcb1692* siderophore production

**Fig 1:**
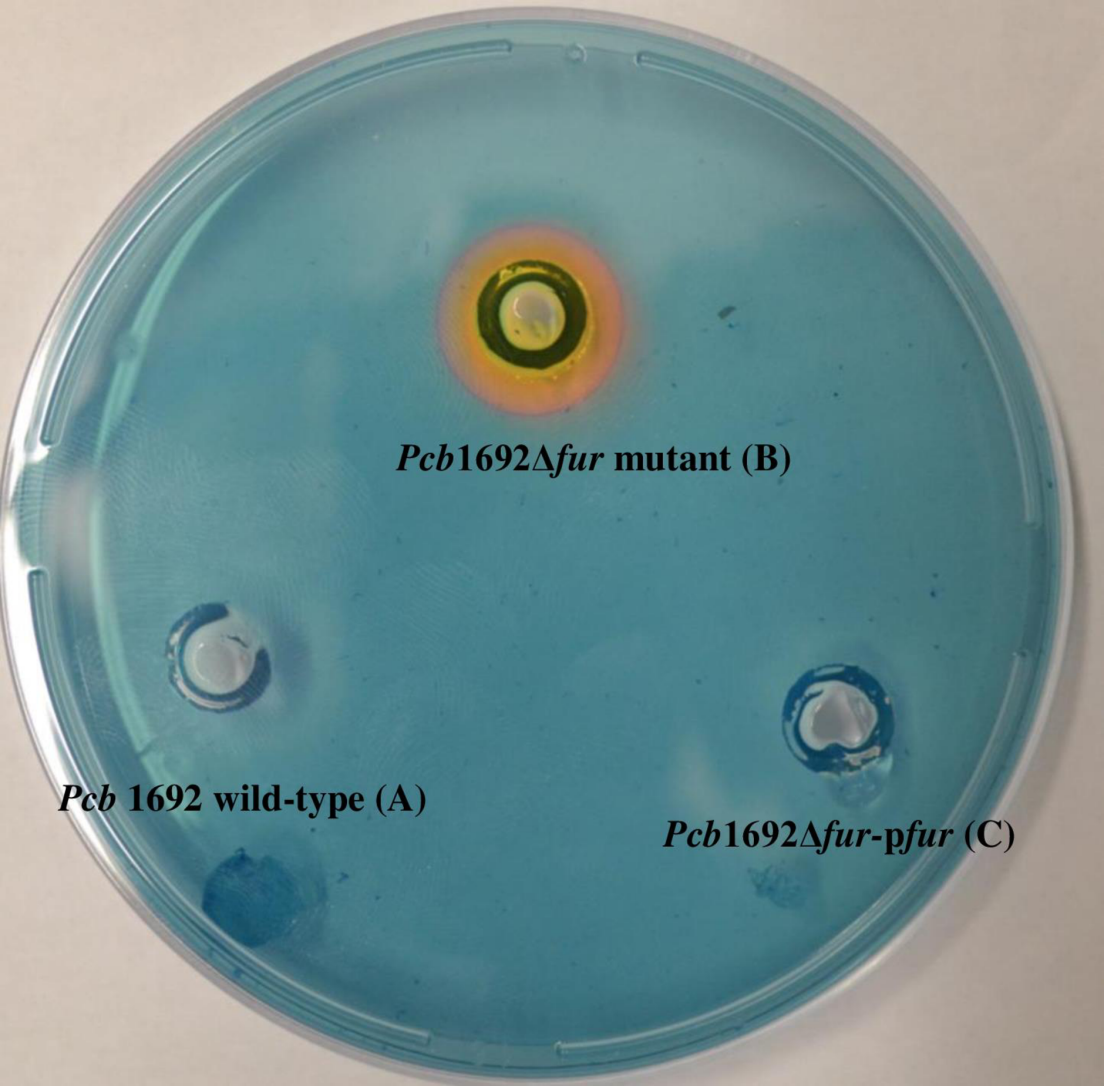
Siderophore production in *Pcb* 1692 wild-type strains compared to *Pcb1692*Δ*fur* mutant strain using Chrome Azurol S (CAS) plate assay. A yellow halo indicates siderophore production. *Pcb* 1692 wild-type (A) and *Pcb1692*Δ*fur*-p*fur* strain (C) showed no visible yellow halo while *Pcb1692*Δ*fur* (B) had a visible yellow halo ring.

The *Pcb*1692Δ*fur* mutant strain showed increased siderophore production relative to the *Pcb1692* wild-type and *Pcb*1692Δ*fur-*p*fur* complemented strain, as indicated by the formation of yellow halos around their colonies (Fig 1). The *Pcb1692* wild-type lacked a yellow halo, suggesting that siderophore production was undetectable under the experimental conditions (Fig 1). The results suggest that the increase in siderophore production in the *Pcb*1692Δ*fur* mutant strain was due to deletion of the *fur* gene.

### Resistance against hydrogen peroxide (H_2_O_2_)

**Fig 2:**
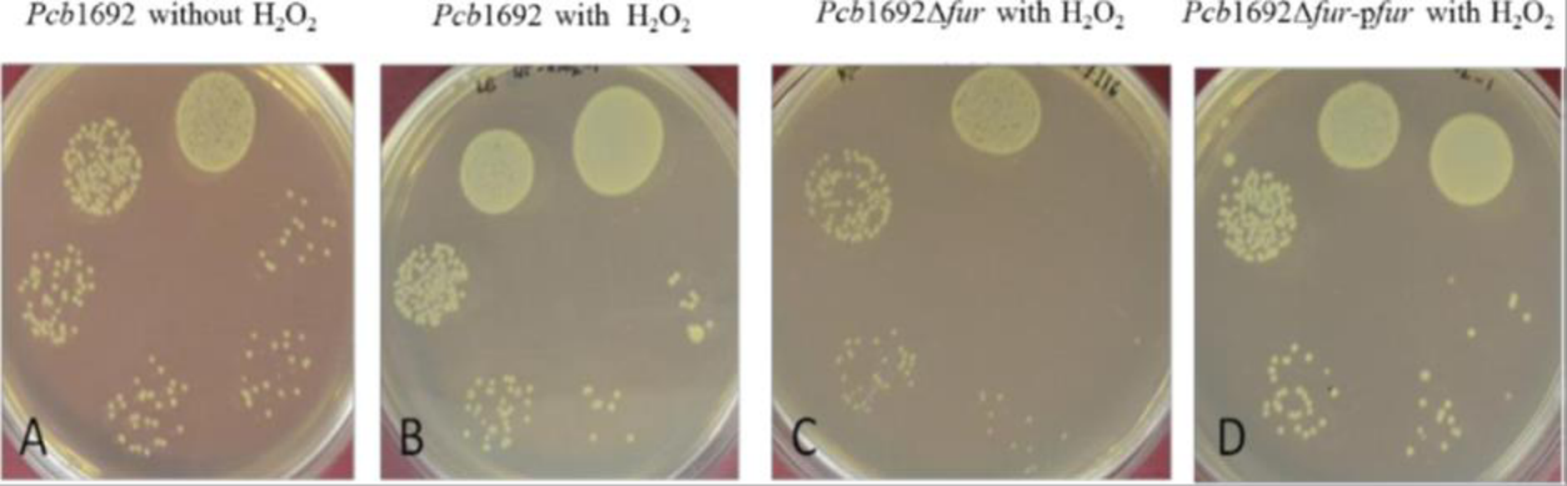
The effect of H_2_O_2_ on *Pcb*1692 wild-type and mutant strain survival. (A) *Pcb* 1692 wild-type cultured in LB medium (B) *Pcb*1692 wild-type in LB medium supplemented with H_2_O_2_ (C) *Pcb1692*Δ*fur* mutant strain cultured in LB supplemented with H_2_O_2_ (D) *Pcb1692*Δ*fur*-p*fur* cultured in LB supplemented with H_2_O_2_.

To evaluate the role of *Pcb1692* Fur protein in the oxidative stress response, bacterial strains were grown in LB broth supplemented with 20μM H_2_O_2_and the bacteria were enumerated (Cfu/ml) after 6 h of incubation. As a control, the *Pcb1692* wild-type strain was grown in LB broth lacking H_2_O_2_. Addition of H_2_O_2_ to the LB broth reduced survival of *Pcb1692* by nearly 10%. Interestingly, there was nearly 70% reduction in survival of the *Pcb*1692Δ*fur* mutant strain when cultured in LB broth supplemented with H_2_O_2_ compared to the wild type under similar conditions (Table 3). Trans-complementation of the *fur* gene in *Pcb*1692Δ*fur* restored survival of the complemented strain to wild type levels. Our results indicate that the *Pcb*1692Δ*fur* mutant strain was more sensitive to H_2_O_2_ than the wild-type strain (Fig 2), suggesting that the Fur protein of *Pcb1692* may play a role in the oxidative stress response in this bacterium.

**Table 3:** Percentage survival of *Pcb1692* wild-type, *Pcb*1692Δ*fur* mutant and *Pcb*1692Δ*fur-*p*fur* complemented strains inoculated into LB broth supplemented with H_2_O_2_ compared to the *Pcb1692* wild-type strain in LB without H_2_O_2_.

Strain	Averaged CFU/ml (6hpi)	Survival (%)
*Pcb1692* wild-type (no H_2_O_2_)	34	100%
*Pcb1692*wild-type (+H_2_O_2_)	31	91.17**±**2.82%
*Pcb*1692Δ*fur* mutant (+H_2_O_2_)	8	23.53**±**1.41%
*Pcb*1692Δ*fur-*p*fur* (+H_2_O_2_)	29	85.29**±**2.82%

### Swimming motility was impaired in the *Pcb*1692Δ*fur* mutant strain

**Fig 3:**
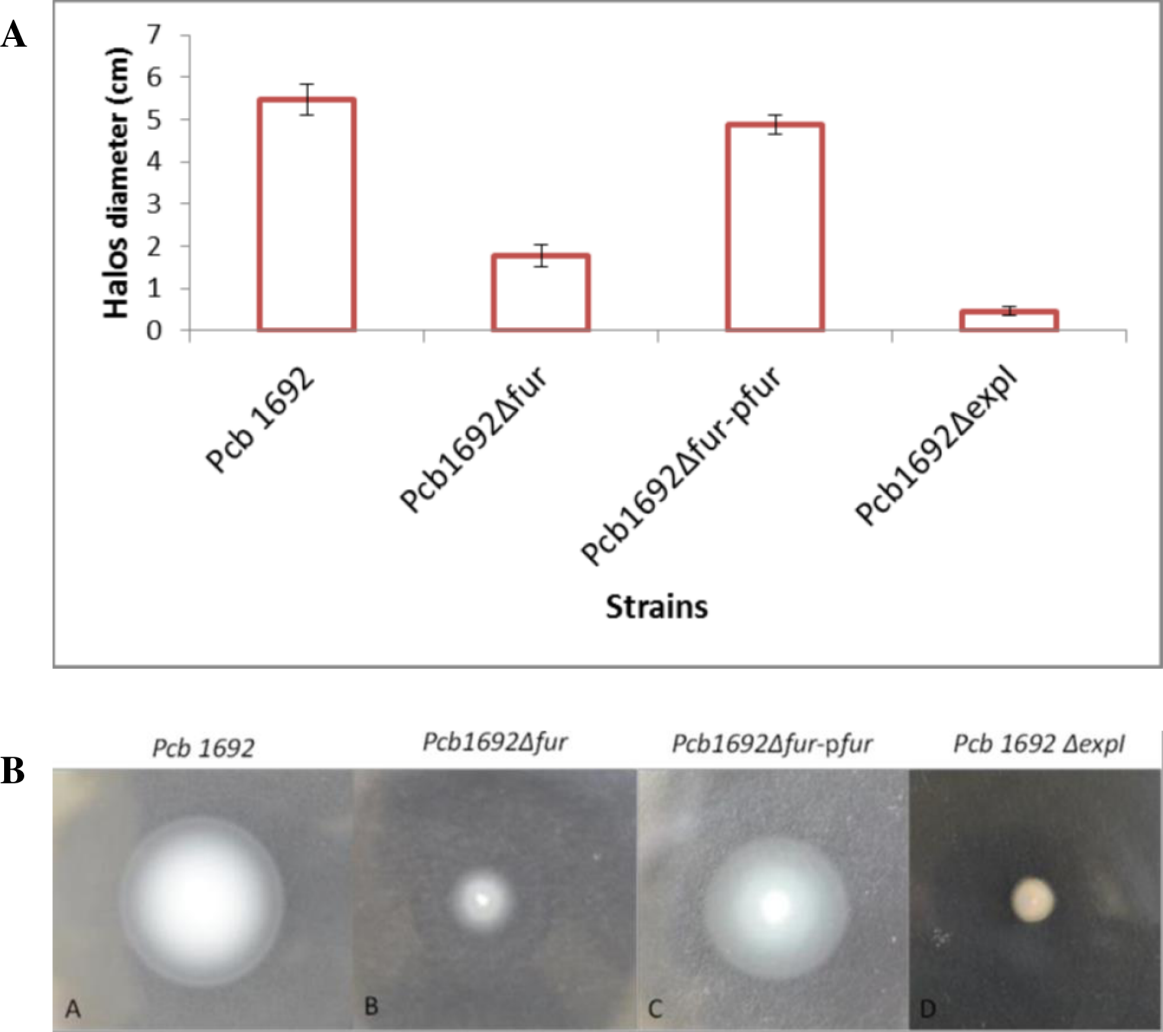
Analysis of swimming motility of the *Pcb1692* wild-type and mutant strain. In the figure, A represents quantitative while B represents qualitative comparison of swimming motility between the wild type and mutant strain. The diameters of halos formed by each strain on LB agar for each strain was measured in triplicates (three biological replicates) and mean values plotted. Error bars represent the standard deviation of the mean. Statistically significant difference were determined by the one way ANOVA and *p*-values less than 0.05 (*p*<0.05) were considered to be statistically significant.

Swimming motility assays indicated that halos formed by the *Pcb1692* wild-type and the complimented *Pcb*1692Δ*fur-*p*fur* strains had diameters of 5.467±0.36cm and 4.867±0.229cm, respectively, at 48 h post-inoculation. On the contrary, the halo diameter of the *Pcb*1692Δ*fur* mutant strain (1.767±0.269cm) was significantly (*p*< 0.05) reduced compared to that of *Pcb1692* wild-type strain (Fig 3A). The *Pcb1692*Δ*expI* mutant strain was used as a negative control since this strain is completely impaired in swimming motility [7]. As expected, the *Pcb1692*Δ*expI* mutant strain showed a complete loss of swimming motility (0.456±0.101 cm) (Fig 3B). Together, our results implicate the *Pcb1692* Fur protein in swimming motility.

### The *Pcb*1692Δ*fur* mutant strain is attenuated in AHL production

**Fig 4:**
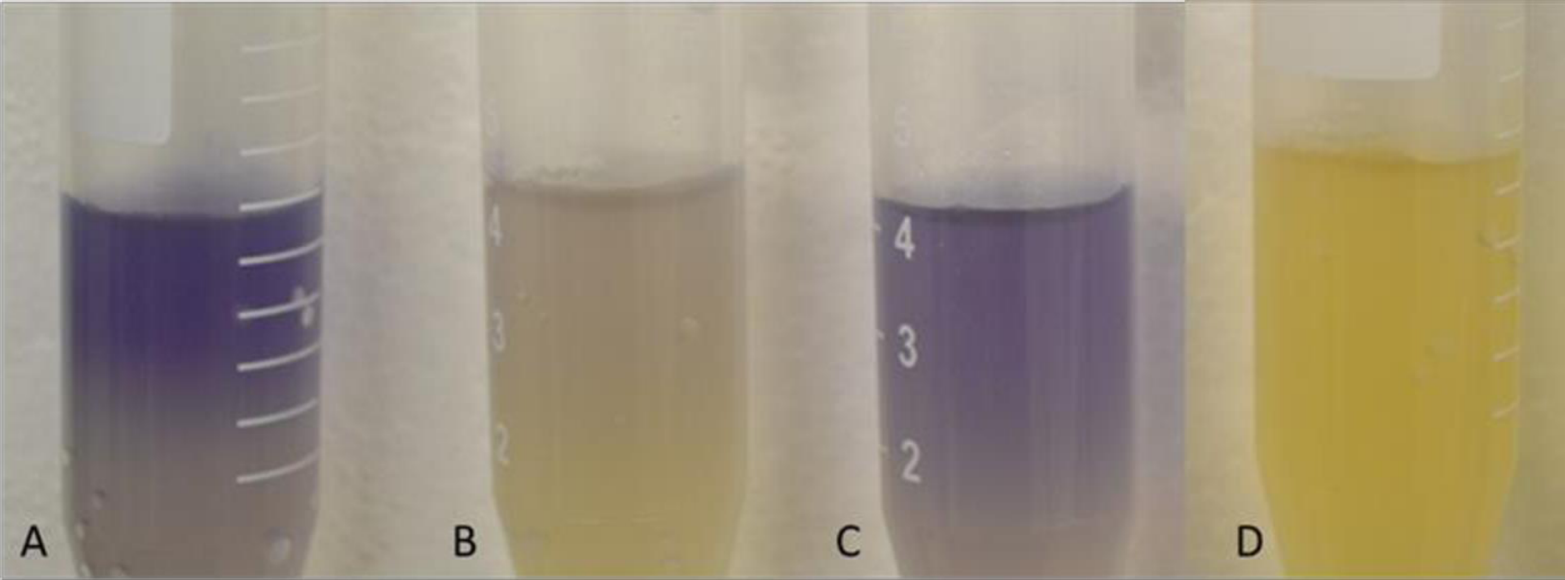
Effect of Fur on synthesis of AHLs in *Pcb1692*Δ*fur* mutant relative to the *Pcb*1692 wild type strain. Production of AHLs was indicated by formation of blue color ring 48 hours after each strain was co-inoculated with CV026 reporter strain at 28°C. (A) *Pcb*1692 wild-type strain (B) *Pcb1692*Δ*fur* mutant (C) *Pcb1692*Δ*fur-*p*fur* complemented mutant strain and (D) *Pcb1692*Δ*expI* mutant strain.

To investigate a role for the *Pcb1692* Fur protein in N-acyl homoserine lactones (AHLs) synthesis, we compared the *Pcb1692*, *Pcb*1692Δ*fur-*p*fur*, *Pcb*1692Δ*fur* and *Pcb1692*0Δ*expI* strains with respect to their ability to synthesize and produce AHLs. To this end, the *C*. *violaceum* CV026 reporter strain was used. As expected, *Pcb1692* (Fig 4A) produced a strong blue colour, indicative of AHL production. On the contrary, the *Pcb*1692Δ*fur* mutant strain produced a faint blue colour, indicating minimal AHL production by this strain. Trans-complementation of the mutant strain with the wild-type *fur* gene in the mutant strain (*Pcb*1692Δ*fur-*p*fur*) restored AHL production (Fig 4C). A *Pcb1692*Δ*expI* mutant strain that lacks the ability to produce AHLs was used as a negative control [7] and accordingly showed no colour change (Fig 4D). These results implicate the *Pcb1692* Fur protein, either directly or indirectly, in AHL production.

### Contribution of Fur in the virulence of *Pcb*1692

**Fig 5:**
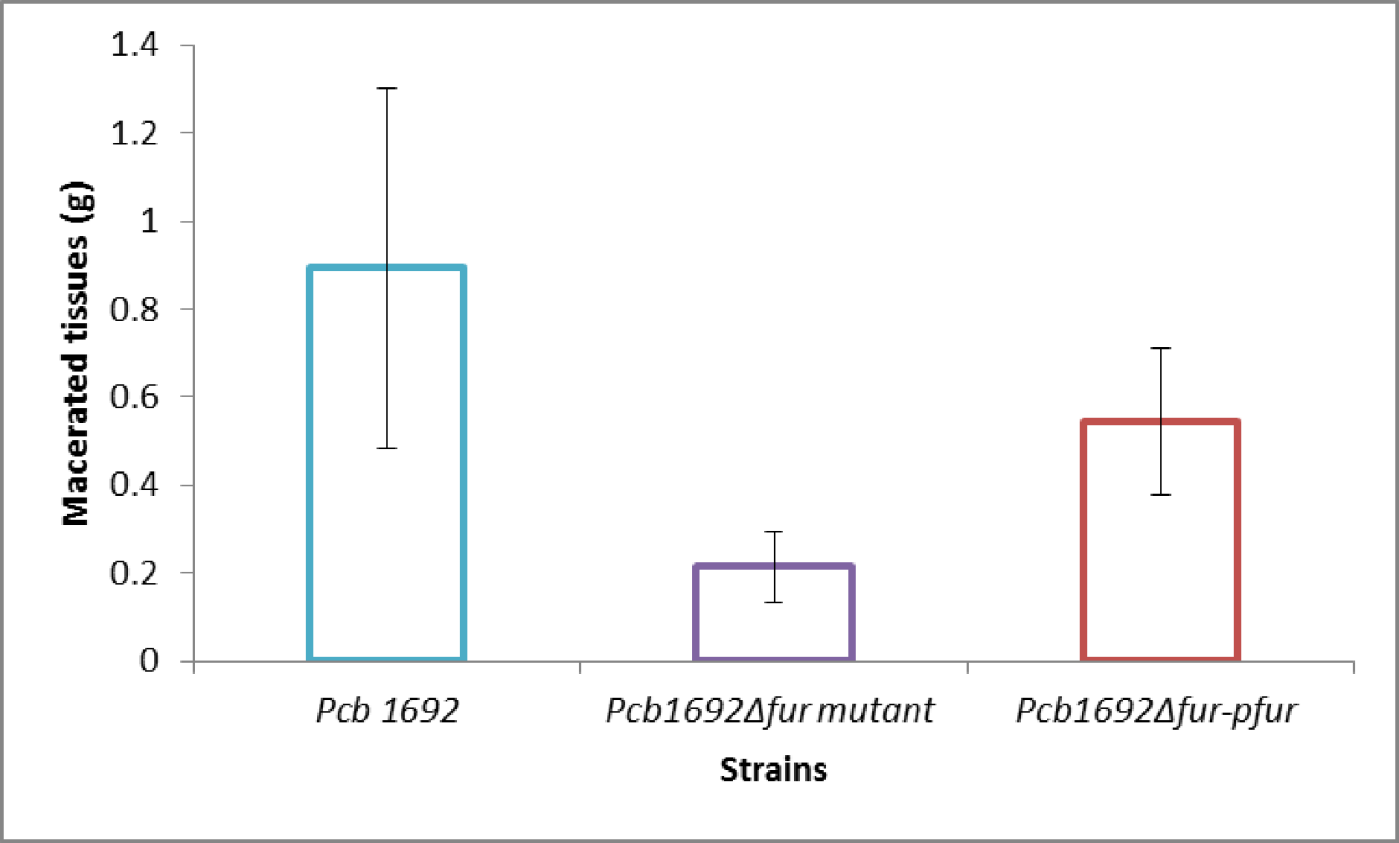
Effect of Fur *in Pcb1692* virulence on potato tubers. Susceptible potato tuber (cv Mondial) were inoculated with *Pcb*1692 wild type strain, *Pcb1692*Δ*fur* mutant and *Pcb1692*Δ*fur*-*pfur* complemented strain (OD_600_ equivalent to 1). MgSO_4_ was used as negative control. Macerated tissue was weighed at 72 hpi.

To determine whether the Fur protein plays a role in the ability of *Pcb1692* to macerate potato tubers, surface-sterilized potato tubers were inoculated with standardized cultures of the*Pcb1692*, *Pcb*1692Δ*fur* and *Pcb*1692Δ*fur-*p*fur* strains. As a control, potato tubers were inoculated with MgSO_4_ buffer. At 72 h post-inoculation, macerated tissue was scooped and weighed. The results showed that the average weight of the macerated tissue due to the*Pcb*1692 wild-type strain was significantly (*p*< 0.05) higher compared to that of the *Pcb*1692Δ*fur* mutant (Fig 5). In addition, trans-complementation of the *fur* gene in the *Pcb*1692Δ*fur* mutant strain (*Pcb*1692Δ*fur-*p*fur* strain) restored the virulence similar to the wild-type strain (Fig 5). Potato tubers mock-inoculated with 10 mM MgSO_4_ showed no tissue maceration. The findings suggest that the Fur regulon of *Pcb*1692 may include several virulence factors involved in maceration of potato tubers.

### Biofilm formation

**Fig 6:**
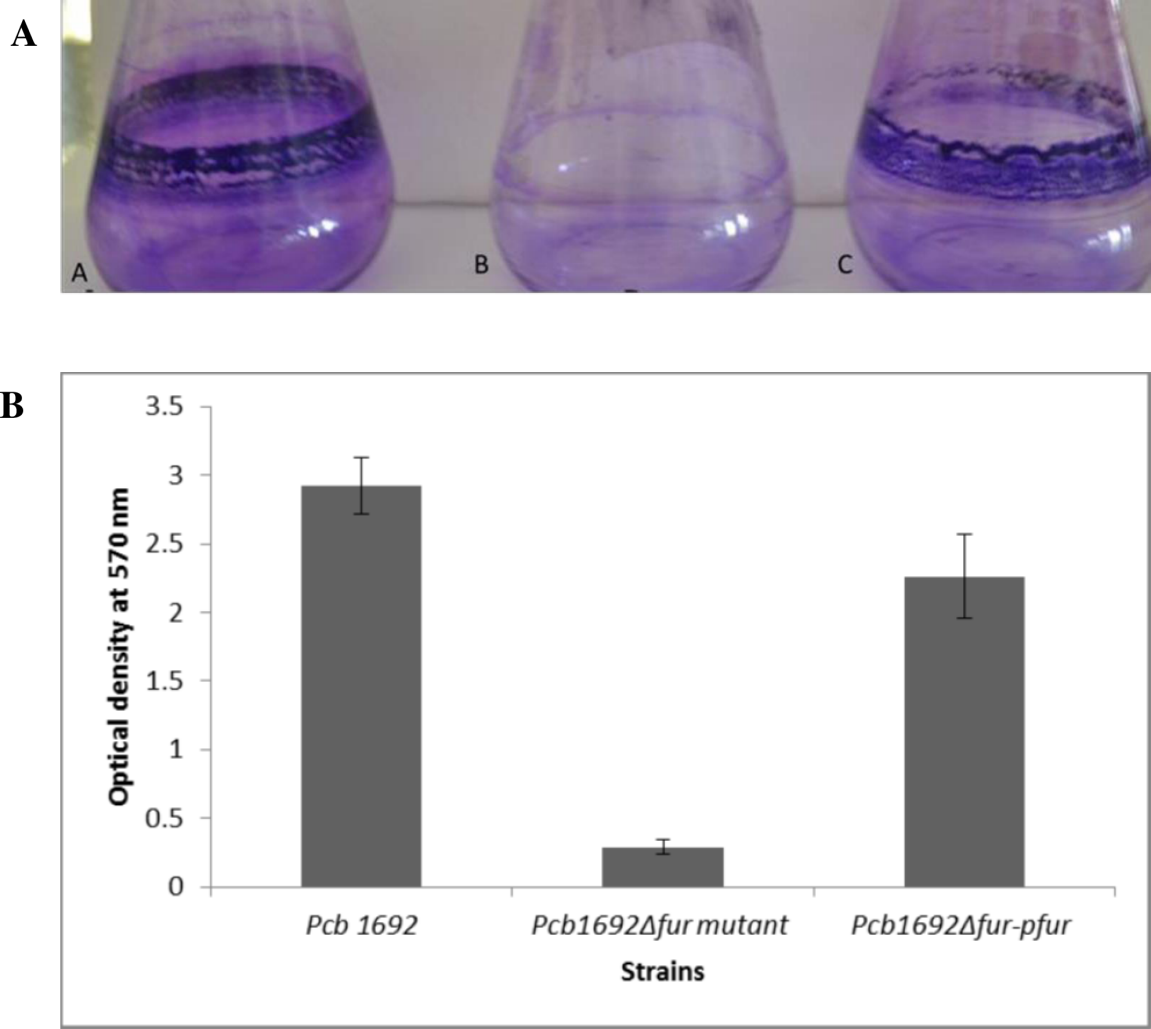
Effect of *fur* mutation on biofilm formation in *Pcb*1692. A. Qualitative biofilm formation showing biofilms in conical flasks after crystal violet staining. Flask A, B, and C represents *Pcb*1692 wild-type, *Pcb1692*Δ*fur* mutant strain, and *Pcb1692*Δ*fur*-p*fur* strain respectively. B. Quantitative biofilm formation by *Pcb*1692 wild-type, *Pcb1692*Δ*fur* mutant and *Pcb1692*Δ*fur*-p*fur* complement strain**.** Standard deviation and averaged mean values represent optical densities (OD_570_) from three independent experiments. Statistically significant difference were determined by the one way ANOVA and *p*-values less than 0.05 (*p*<0.05) were considered to be statistically significant.

Biofilm formation is an important virulence determinant of many phytopathogens and animal bacterial pathogens. In *Pcb*, biofilm formation may contribute to the colonization of potato tubers and eventually disease development. We thus investigated whether the Fur protein affects biofilm formation by *Pcb1692* using crystal violets (CV) staining assay. The results indicated that the*Pcb*1692 wild-type and *Pcb*1692Δ*fur-*p*fur* complemented strains both formed a biofilm on the inner surface of a conical flask, whereas the *Pcb*1692Δ*fur* mutant did not form a biofilm under the same assay conditions (Fig 6A). These results were further confirmed with a quantitative biofilm assay, the results of which demonstrated that the absorption mean value of the biofilm by the *Pcb*1692Δ*fur* mutant was significantly reduced (*p*<0.05) with 2 fold reduction compared to those of the *Pcb1692* wild-type and *Pcb*1692Δ*fur-*p*fur* strains (Fig 6B, Table 4).

**Table 4:** Quantitative biofilm formation between *Pcb1692* wild-type, *Pcb*1692Δ*fur* mutant and *Pcb*1692Δ*fur-*p*fur* complement strain. Standard deviation and averaged mean values represent optical densities (OD_570_) from three independent experiments. Statically significant differences were determined by the one way ANOVA and *p*-values less that 0.05 (*p*<0.05) were considered to be statistically significant (*p*<0.05).

Strain	Optical Density (OD_570_)
*Pcb1692* wild-type	2.92± 0.204
*Pcb*1692Δ*fur* mutant	0.289± 0.052
*Pcb*1692Δ*fur-*p*fur*	2.26± 0.307

### *The Pcb*1692Δ*fur* mutant strain is attenuated in extracellular enzyme production

**Fig 7:**
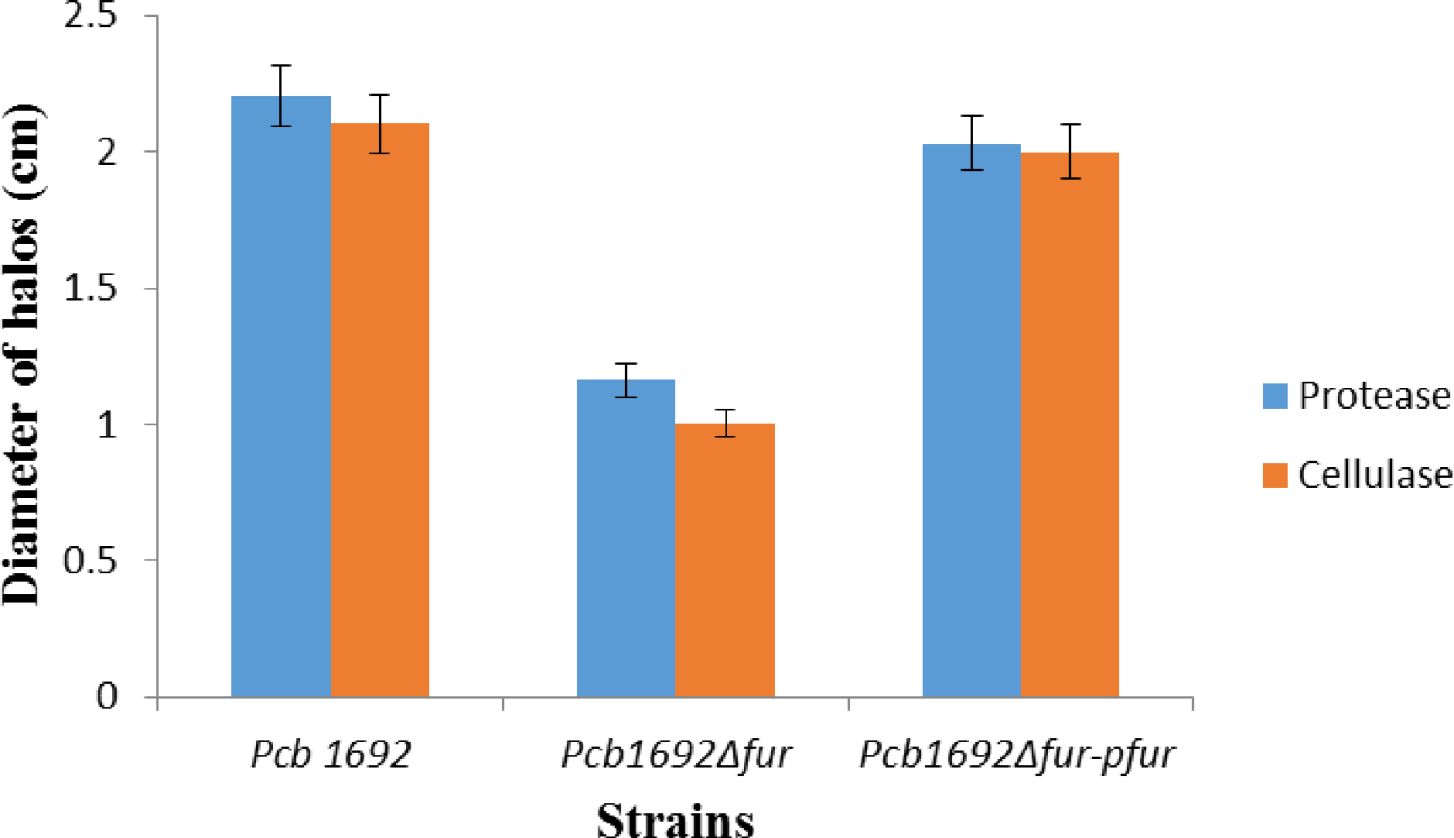
Quantitative cellulase and protease activity of *Pcb*1692, *Pcb1692*Δ*fur* mutant and *Pcb1692*Δ*fur*-p*fur* complement strain. Halo diameter in *Pcb1692*Δ*fur* was significantly reduced compared to *Pcb1692* wild-type and *Pcb1692*Δ*fur*-p*fur* (*p*<0.05).

*Pcb1692*, like other brute-force pathogens, secretes several plant cell wall-degrading enzymes (PCWDEs) that macerate plant tissues, thereby releasing nutrients and iron. Given that Fur regulates iron homeostasis and secretion of some PCWDEs in soft rot Enterobacteriaceae [16], we reasoned that the reduced virulence associated with the *Pcb*1692Δ*fur* mutant strain in potato tubers may be associated with reduced synthesis and secretion of PCWDEs. To test this hypothesis, we assayed the production of two different PCWDEs, namely cellulase and protease, as described in the Materials and Methods. Consistent with our hypothesis, the results showed that both the cellulase and protease activities were significantly reduced in the *Pcb*1692Δ*fur* mutant strain compared to the *Pcb1692* wild-type and *Pcb*1692Δ*fur-*p*fur* complemented strains, thus implicating Fur, directly or indirectly, in the synthesis and production of some PCWDEs in *Pcb1692* (Fig 7).

### Involvement of Fur in *Pcb*1692 EPS production

**Fig 8:**
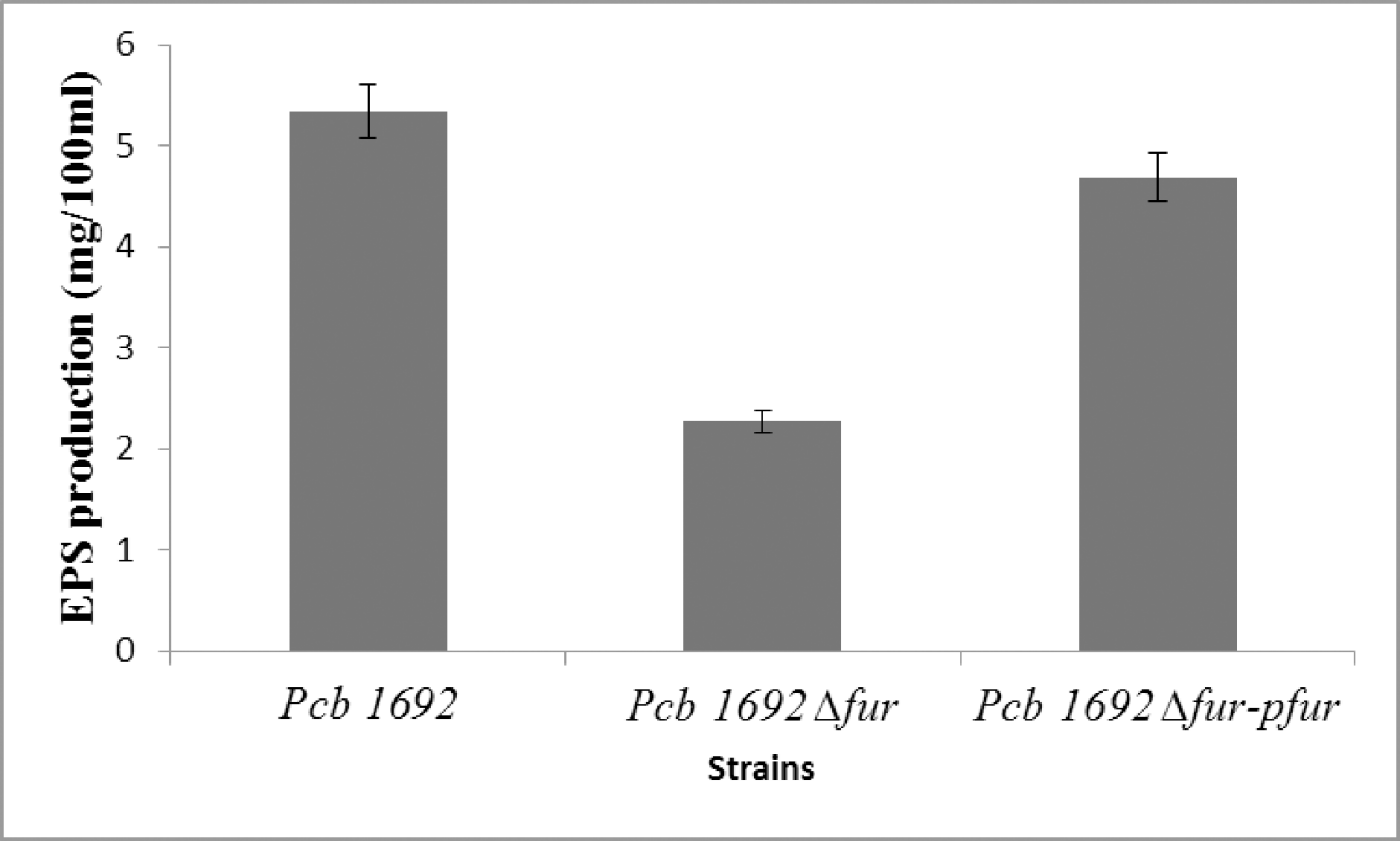
Comparison of extracellular EPS production by *Pcb1692*Δ*fur* and *Pcb*1692 wild type strains. EPS production in *Pcb1692*Δ*fur* was significantly reduced compared to *Pcb1692* wild-type and *Pcb1692*Δ*fur*-p*fur* (*p*<0.05). The error bars represent EPS mean values from the three experiments and three biological experiments.

*Pcb*1692 causes stem rot and eventual wilting of potato plants [2]. This phenomenom has been associated with the production of copious amounts of EPS which occludes xylem vessels, resulting in wilting and die-back of plants [38]. In addition, EPS is a major component of biofilms that aids in protecting bacteria from UV light, plant antimicrobial agents, and serves as a source of nutrients to the bacteria, thus making EPS production an important virulence determinant in several bacteria, including phytopathogens [38]. EPS production was determined by weighing the amount of EPS produced by the wild-type, *Pcb*1692Δ*fur* and *Pcb*1692Δ*fur-*p*fur* strains grown in LB broth for 72 h. Our results showed that wild-type *Pcb*1692 produced 5.34 mg/100ml of EPS compared to 2.27 mg/100ml for *Pcb*1692Δ*fur* mutant and 4.69 mg/100ml for the complemented *Pcb*1692Δ*fur-*p*fur* strain (Fig 8). These findings implicate the *Pcb1692* Fur protein in the regulation of EPS production.

### Gene expression

Given that Fur has been implicated in the regulation of bacterial genes associated with virulence, and fitness [36], we next investigated a role for the *Pcb1692* Fur protein in regulating the expression of randomly selected virulence and fitness genes. The genes selected in this study have been shown previously to be under regulation the Fur protein. The genes selected for qRT-PCR included: *AED-0004132* (encoding ferredoxin), *hasA* (encoding an extracellular heme-binding protein) and *sodC* (encoding a copper-zinc superoxide dismutase). We also included genes encoding PCWDEs such as *prtA* (encoding a protease) and *celV* (encoding cellulase), as well as *motA* (encoding the flagellar motor protein) and *fliC* (encoding a flagellar transcriptional activator).

**Fig 9:**
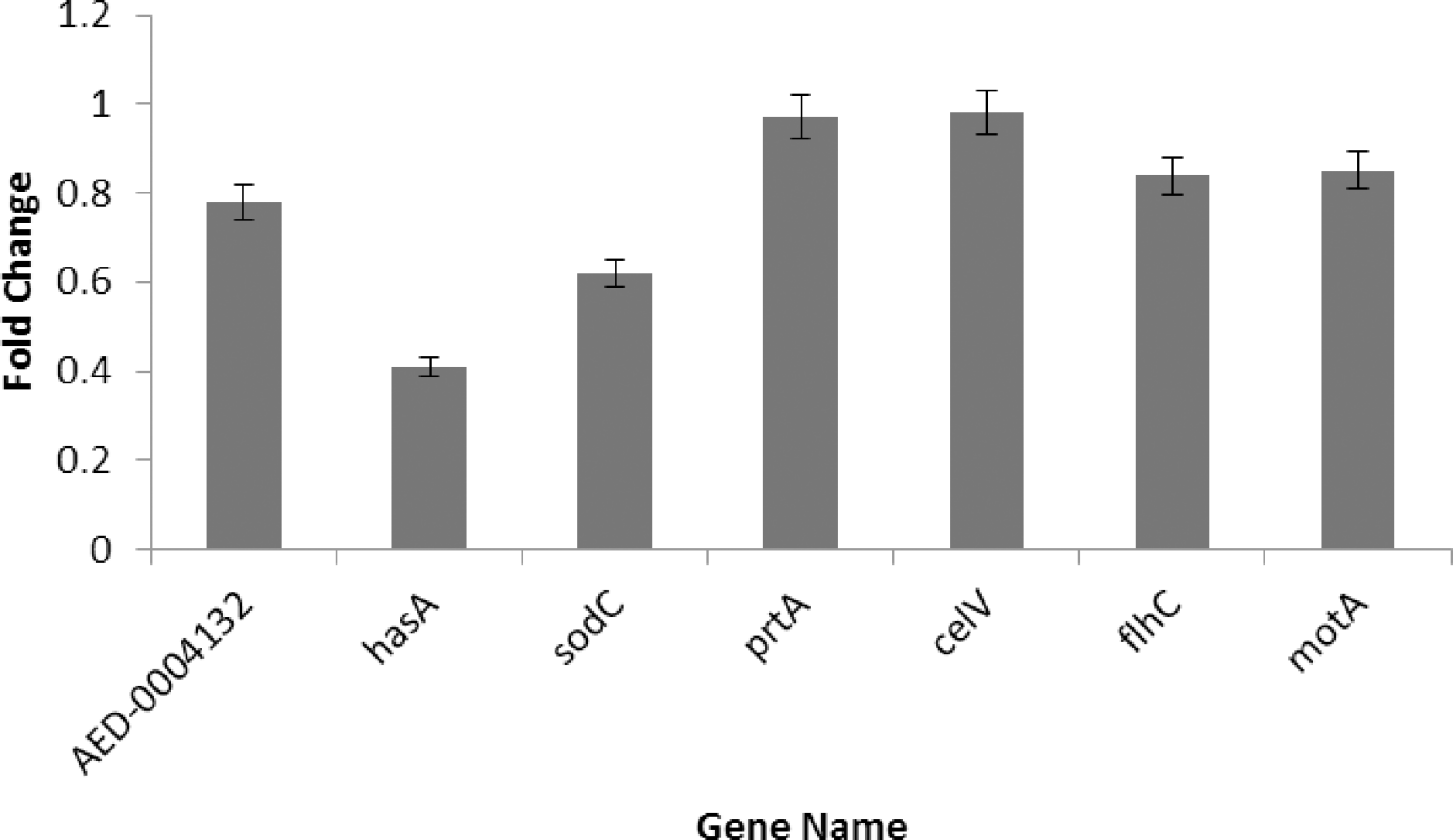
Differentially expressed candidate pathogenicity genes involved in iron uptake. (*AED-004132*, *hasA*), oxidative stress tolerance (*sodC*), plant cell wall degrading enzymes (*prtA*, *celV*) and motility (*flhC*, *motA*) in *Pcb1692*Δ*fur* mutant strain relative to *Pcb*1692 wild-type. The *ffh* gene encoding signal recognition particle subunit was used as housekeeping gene to normalize gene expression.

The qPCR results indicated that the relative expression of *AED-0004132*, *hasA* and *sodC* was significantly reduced in the *Pcb*1692Δ*fur* mutant strain (0.78-, 0.41- and 0.62-fold, respectively) compared to the *Pcb1692* wild-type strain. These findings confirm that the Fur protein of *Pcb1692* is involved in iron homeostasis and the stress response. In addition, *motA* (0.84-fold) and *flhC* (0.85-fold) were significantly down-regulated in the *Pcb*1692Δ*fur* mutant strain compared to *Pcb*1692 wild-type, indicating that expressions of some flagella-related genes are also regulated by Fur (Fig 9). Conversely, both the *celV* and *prtA* gene expression levels were significantly reduced in the *Pcb*1692Δ*fur* mutant strain (0.97-and 0.98-fold, respectively) compared to *Pcb*1692 wild-type confirming that Fur regulates production of some PCWDEs based on our results.

### Putative ‘fur boxes’ identified upstream of a selected number of genes in *Pcb1692*

In an attempt to establish whether the observed phenotypes of Fur mutant relative to the wild type strain are indeed a consequence of Fur regulation, we searched for presence of fur boxes upstream of a selected number of genes responsible for some of the observed phenotypes. Our bioinformatics analysis indicated that genes regulated by Fur contained a GATAAT iron binding sequence indicated by red boxes (S3 Fig). These sequences were all found upstream of the selected genes within or near the promoter regions. The consensus sequence shows that at least one repeat of conserved GATAAT sequence is present within the selected genes with AT rich regions prior to/or following the repeat. The exact GATAAT hexameric repeat and corresponding palindrome sequences were not identified here.

## Discussion

The function of the ferric uptake regulator (Fur) protein has been studied in a number of bacteria, including plant pathogens such as *Erwinia chrysanthemi*, *Pantoea sterwartii*, *Xanthomonas vesicatoria* and *Xanthomonas campestris* pv. *campestris* [15, 16, 39, 40]. However, the role of *fur* in *Pcb*1692, an important emerging pathogen of potatoes and other crops, has not been investigated before. In addition, the mode of action of the Fur protein has been extensively studied in *E*. *coli*, where it has been shown that in the presence of ferric ion (Fe^2+^), the Fur protein binds and forms a complex with Fe^2+^ [25]. The resulting Fur-Fe^2+^ complex binds to a conserved consensus sequence called the ‘*fur* box’, which is located within the promoter region of many genes. This binding results in transcriptional repression of genes involved in iron acquisition and storage [25]. Given that Fur cannot bind to the ‘fur box’ sequence in the absence of iron, transcriptional repression of target genes is relieved [41].

In the current study, we deleted the *fur* gene from the genome of *Pcb*1692 and functionally characterized its role in virulence, fitness and host-pathogen interactions. Our data demonstrated that siderophore production was undetectable in *Pcb*1692 wild-type, while the *Pcb*1692Δ*fur* mutant strain produced statistically significant higher levels of siderophore. These findings demonstrate that Fur of *Pcb1692* negatively regulates the synthesis and production of siderophores. Siderophores are iron scavengers that are produced by many Gram-negative bacteria as a crucial means of acquiring iron in iron-limiting environments [42]. Consistent with our findings, deletion of the *fur* gene in many bacteria, including *X*. *vesicatoria*, resulted in an increased production of siderophores [39]. In addition, the *Pcb*1692Δ*fur* mutant was more sensitive to oxidative stress and was attenuated in EPS production, biofilm formation, swimming motility and virulence in potato tubers. Together, these findings indicate that the Fur protein of *Pcb1692* plays a vital role in regulating many virulence factors, directly or indirectly.

Fur plays a pivotal role in preventing damage caused by the production of reactive oxygen species (ROS) within the host plants by regulating genes involved in reactive oxygen detoxification [14]. In many Gram-negative bacteria, deletion of the *fur* gene results in cell death due to oxidative stress and damage [19]. Consistent with these findings, our results indicated that survival of the *Pcb*1692Δ*fur* mutant strain was severely reduced compared to the *Pcb1692* wild-type strain and the complemented strain *Pcb*1692Δ*fur*-p*fur* when cultured in the presence of 20μM of H_2_O_2_. These findings demonstrate that the *Pcb*1692Δ*fur* mutant strain was impaired in its ability to resist oxidative stress and damage. It is possible that the *Pcb*1692Δ*fur* mutant strain may have lost the ability to regulate genes involved in degradation of ROS. This hypothesis is supported by gene expression analysis, which showed that the *sodC* gene (encoding for superoxide dismutase) was significantly down-regulated in the *Pcb*1692Δ*fur* mutant strain compared to the wild-type (Fig 9).

Motility is an important virulence determinant during the epiphytic and invasion stages in many pathogenic bacteria, including *Pectobacterium carotovorum* subsp. *carotovorum*, and enables bacterial cells to swim to nutrient-rich niches or avoid environmental stresses [43]. Moleleki and colleagues observed that quorum sensing-defective *Pcb*1692 was not motile and suggested that quorum sensing regulates genes involved in flagella synthesis [7]. Our results demonstrated that motility of the *Pcb*1692Δ*fur* mutant strain was significantly reduced compared to the wild-type strain, suggesting that the *Pcb*1692 Fur protein may be involved in regulating motility. Consistent with these findings, gene expression levels of *flhC* and *motA* were significantly reduced in the *Pcb*1692Δ*fur* mutant strain compared to the wild-type strain. It is currently not clear how Fur regulates flagella and quorum sensing-related genes in *Pcb1692* and therefore, needs to be investigated in the future studies.

Iron acquisition has been shown to be an important virulence factor for many pathogenic bacteria; iron uptake is tightly regulated by the ferric uptake regulator (*fur*) [14]. In this study we found that the *Pcb1692*Δ*fur* mutant strain was significantly reduced in virulence compared to the wild-type strain, suggesting that Fur contributes to *Pcb1692* virulence on potato tubers. We hypothesized that disrupting the *fur* gene, which is a metal-sensing system in *Pcb*, resulted in high uptake of iron thereby causing toxicity and hence, cell death. High intracellular concentrations of iron results in the generation of reactive oxygen species, causing oxidative stress and eventually damage cells [14]. Previous studies have shown that mutation of the *fur* gene in *X*. *vesicatoria* and *X*. *campestris* pv. *campestris* resulted in reduced virulence, biofilm formation, EPS and AHL production; similar to the results observed for the *Pcb*1692Δ*fur* mutant in this study [16, 39]. Expression of the *AED-0004132* (ferredoxin) and *hasA* (extracellular heme-binding protein) genes, which are involved in iron uptake, were significantly down-regulated in the *Pcb*1692Δ*fur* mutant strain. It is tempting to speculate that the Fur protein of *Pcb1692* is may therefore be an important regulator of iron uptake during *in planta* infection and may be involved directly or indirectly in regulating other virulence factors such as motility, which is important for successful pathogenesis. These findings strongly suggest that Fur is more likely to be a regulator of the expression of virulence determinants in *Pcb1692*.

Production of PCWDEs such as proteases, cellulases and pectinases by many phytopathogens has been shown to be important for plant disease symptom development [10]. In *Pectobacterium atrosepticum* (*Pba*), production and secretion of PCWDEs and other virulence factors is tightly regulated by a quorum sensing mechanism [39]. Quorum sensing is only one of several complex regulatory networks that modulate the expression of virulence factors in bacteria. Lamont and colleagues showed that iron availability not only controls the production and secretion of siderophores, but also regulates production of PCWDEs (protease) and antibiotics in *P*. *aeruginosa* [44]. It is interesting to note that production of cellulase and proteases were significantly lower in *Pcb*1692Δ*fur* compared to the wild-type strain and the complemented *Pcb*1692Δ*fur*-p*fur* strain. These phenotypes were confirmed using gene expression analysis, which demonstrated that the *celV* and *prtA* expression levels were significantly reduced in the *Pcb*1692Δ*fur* mutant compared to the *Pcb*1692 wild-type strain. A link between quorum sensing and the Fur protein was investigated by evaluating the production of N-acyl homoserine lactones (AHLs) in a quorum sensing-deficient mutant (*Pcb*1692Δ*expI*) and the *Pcb1692*Δ*fur* mutant strain. It was observed that while *Pcb*1692Δ*expI* completely lacked the ability to produce AHLs, the *Pcb*1692Δ*fur* mutant displayed reduced production of AHLs compared to the *Pcb*1692 and *Pcb*1692Δ*fur*-p*fur* strains. This, it appears that Fur may be directly or indirectly involved in the regulation of virulence factors under quorum sensing control.

The Fur protein in *Escherichia coli* has been well characterized and shown to act as a transcriptional repressor to iron-related genes by binding to ‘fur boxes’ found in the promoter region [22]. The complex prevents the entry of RNA polymerase therefore inhibiting initiation of transcription [22]. Our bioinformatics analysis revealed predicted ‘fur boxes’ in a number of genes involved in iron uptake, oxidative stress, motility and plant cell wall degrading enzymes. These results suggested that Fur protein in *Pcb1692* represses not only iron-regulated genes but many genes coding for other virulence factors. Indicating *fur* regulon in *Pcb1692* plays a more important role in virulence than previously thought. This argument is supported by results obtained by Franza and colleagues where they found that some genes coding for PCWDEs harbour ‘fur boxes’ [16].

In conclusion, *Pcb*1692 *fur* gene was characterized and shown to regulate the expression of genes involved in iron acquisition and iron storage systems. Regulation of iron uptake could be pivotal for survival and virulence of *Pcb*1692 in potato tubers, given that in the early stages of infection iron is limiting in this environment and *Pcb*1692 must therefore compete with other bacteria for the limited iron. At the later stage of infection when *Pcb*1692 reaches a high cell density (quorum) the pathogen synthesizes and secretes PCWDEs which macerate potato tubers thereby releasing nutrients and iron. It may therefore be pivotal for *Pcb*1692 to coordinate production of PCWDE with availability of iron. Conversely, the presence of excess iron leads to oxidative burst generated from the Fenton reaction. To survive this oxidative stress *Pcb*1692 produces proteins such as superoxide dismutase (SodC) which neutralize the deleterious effects of the reactive oxygen species. Furthermore, when nutrients and iron are depleted from macerated tissue the *Pcb*1692 Fur protein then, directly, or indirectly up-regulates flagella genes allowing the pathogen to move to a nutrient rich environment. Together, the data presented here shows that the *Pcb*1692 Fur regulon is not limited to iron metabolism but also regulates genes which encode protein associated with virulence factors, motility, oxidative stress and quorum sensing.

## Supporting information

S1 Fig Schematic representation of how *Pcb1692*Δ*fur* mutant strain was generated. A) Using specific set of primers, PCR amplifications of the *fur* upstream and downstream regions were generated as indicated in S1A Fig. Kanamycin cassette was amplified from pKD4 plasmid with primers Kan F and Kan R. B) Primers Fur F and R, were used in a PCR reaction consisting of, the *fur* upstream kanamycin and downstream PCR fragment to generate a PCR fusion product. C) The fusion product was electroporated into electrocompetent *Pcb*1692 to generate the *Pcb1692*Δ*fur* mutant strain (S1D Fig). Both electrocompetent *Pcb*1692 and *Pcb1692*Δ*fur* mutant strain were electroporated with empty pTrc99A.
(TIF)

S2 Fig PCR amplicons used to generate the *fur* mutant. Lane 1. DNA ladder, 2. *fur* downstream PCR fragment, 3. kanamycin cassette PCR product, 4. *fur* upstream PCR fragment, 5. Fusion product consisting of the downstream, kanamycin and upstream fragment. 6. The fragment used for complementation which contains the *fur* gene and its promoter region. 7. Control.
(TIF)

S3 Fig Schematic presentation of the putative promoter regions of selected genes in *Pcb*1692. Based on our qRT-PCR results, some of the genes under the *Pcb*1692 Fur regulon were aligned to the consensus fur box and the putative fur boxes for each gene is indicated by red boxes.
(TIF)
