# Supplementary figures and images for: Influence of the ferric uptake regulator (Fur) protein on pathogenicity in Pectobacterium carotovorum subsp. brasiliense
Source: PLoS One. 2017 May 17;12(5):e0177647. doi: 10.1371/journal.pone.0177647 (PMC5435245; doi:10.1371/journal.pone.0177647)

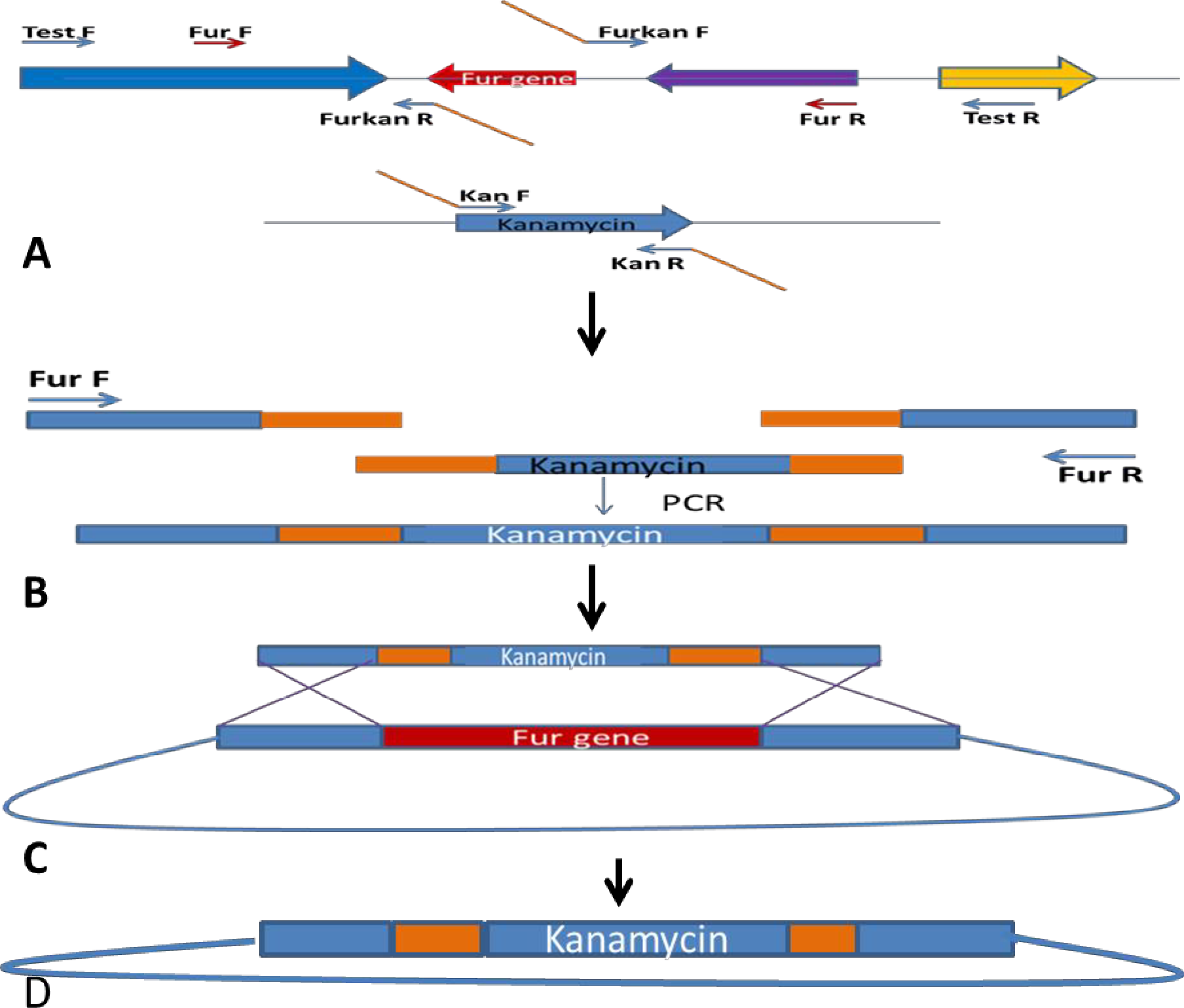

Supplement: S1 Fig — A) Using specific set of primers, PCR amplifications of the fur upstream and downstream regions were generated as indicated in S1A Fig. Kanamycin cassette was amplified from pKD4 plasmid with primers Kan F and Kan R. B) Primers Fur F and R, were used in a PCR reaction consisting of, the fur upstream kanamycin and downstream PCR fragment to generate a PCR fusion product. C) The fusion product was electroporated into electrocompetent Pcb1692 to generate the Pcb1692Δfur mutant strain (S1D Fig). Both electrocompetent Pcb1692 and Pcb1692Δfur mutant strain were electroporated with empty pTrc99A. (TIF) [file pone.0177647.s001.tif]

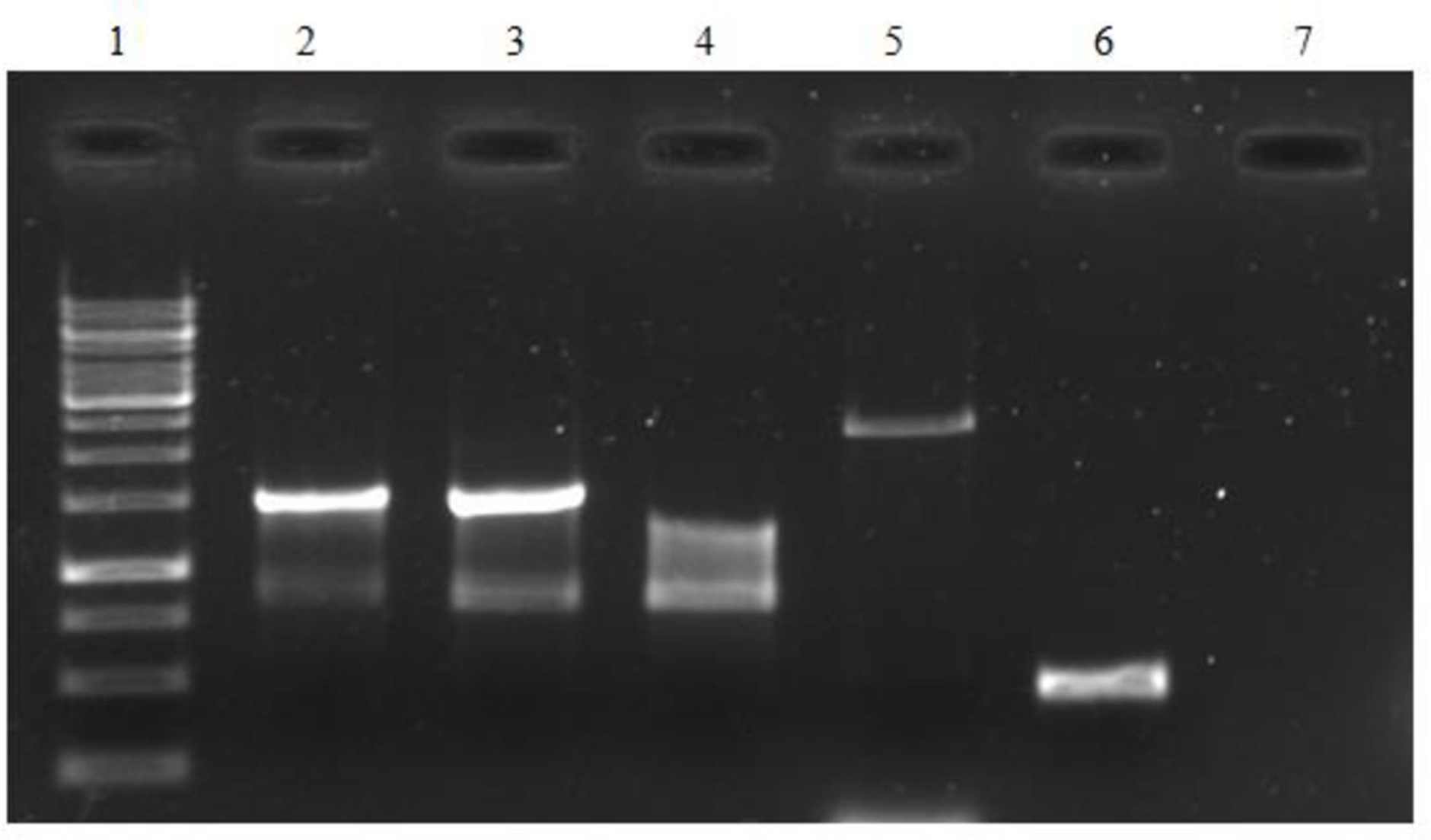

Supplement: S2 Fig — Lane 1. DNA ladder, 2. fur downstream PCR fragment, 3. kanamycin cassette PCR product, 4. fur upstream PCR fragment, 5. Fusion product consisting of the downstream, kanamycin and upstream fragment. 6. The fragment used for complementation which contains the fur gene and its promoter region. 7. Control. (TIF) [file pone.0177647.s002.tif]

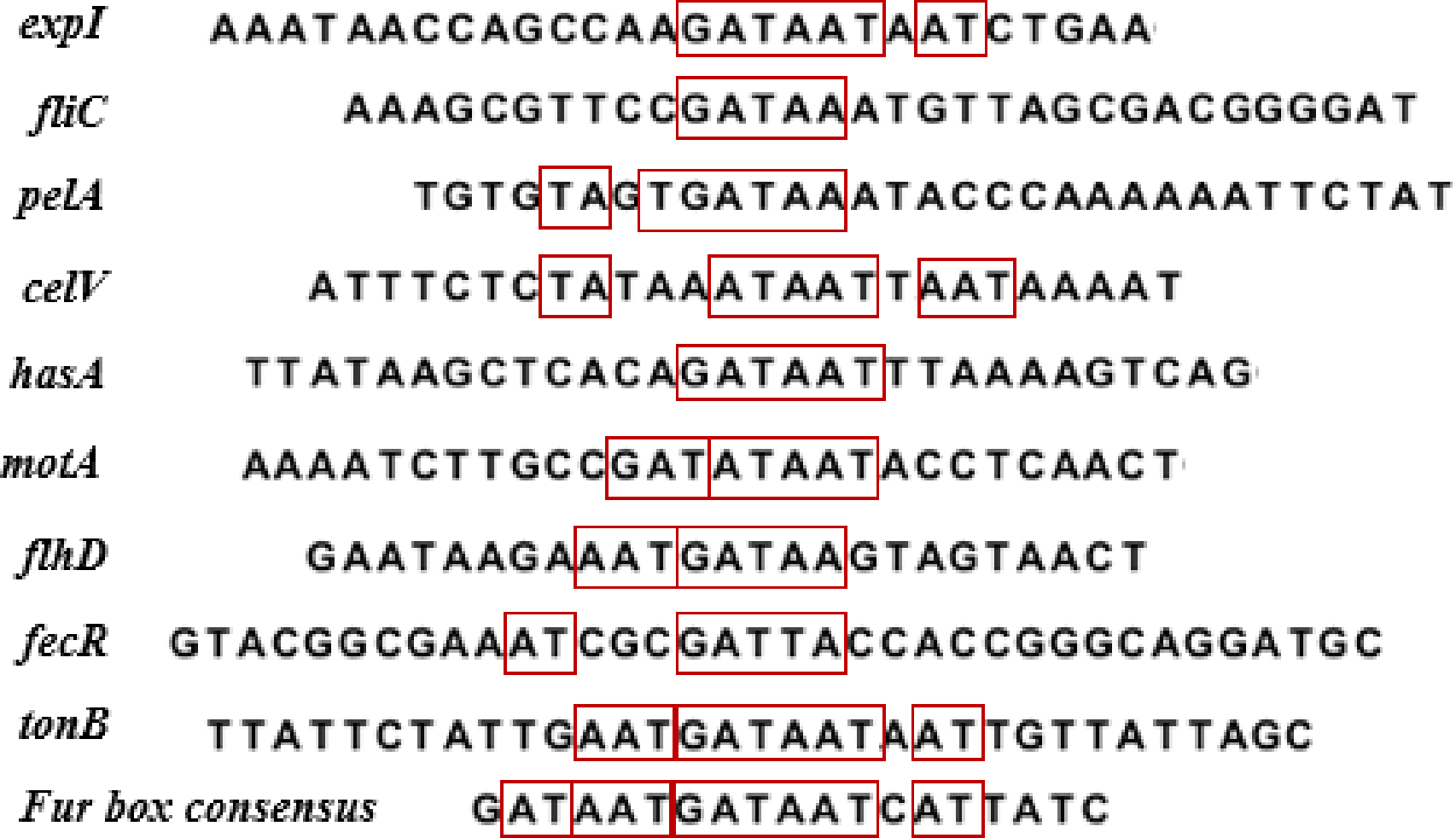

Supplement: S3 Fig — Based on our qRT-PCR results, some of the genes under the Pcb1692 Fur regulon were aligned to the consensus fur box and the putative fur boxes for each gene is indicated by red boxes. (TIF) [file pone.0177647.s003.tif]
